# Detection of transcriptional difference of porcine imprinted genes using different microarray platforms

**DOI:** 10.1186/1471-2164-7-328

**Published:** 2006-12-28

**Authors:** Shengdar Tsai, Bashir Mir, Amy C Martin, Jose L Estrada, Steve R Bischoff, Wen-ping Hsieh, Joseph P Cassady, Bradley A Freking, Dan J Nonneman, Gary A Rohrer, Jorge A Piedrahita

**Affiliations:** 1 Department of Molecular Biomedical Sciences, College of Veterinary Medicine, North Carolina State University, Raleigh, NC, USA; 2 USDA/ARS, U.S. Meat Animal Research Center, Clay Center, NE, USA; 3 Center for Comparative Medicine and Translational Research, North Carolina State University, Raleigh, NC, USA; 4 Bioinformatics Research Center, North Carolina State University, Raleigh, NC, USA

## Abstract

**Background:**

Presently, multiple options exist for conducting gene expression profiling studies in swine. In order to determine the performance of some of the existing microarrays, Affymetrix Porcine, Affymetrix Human U133+2.0, and the U.S. Pig Genome Coordination Program spotted glass oligonucleotide microarrays were compared for their reproducibility, coverage, platform independent and dependent sensitivity using fibroblast cell lines derived from control and parthenogenic porcine embryos.

**Results:**

Array group correlations between technical replicates demonstrated comparable reproducibility in both Affymetrix arrays. Glass oligonucleotide arrays showed greater variability and, in addition, approximately 10% of probes had to be discarded due to slide printing defects. Probe level analysis of Affymetrix Human arrays revealed significant variability within probe sets due to the effects of cross-species hybridization. Affymetrix Porcine arrays identified the greatest number of differentially expressed genes amongst probes common to all arrays, a measure of platform sensitivity. Affymetrix Porcine arrays also identified the greatest number of differentially expressed known imprinted genes using all probes on each array, an *ad hoc *measure of realistic performance for this particular experiment.

**Conclusion:**

We conclude that of the platforms currently available and tested, the Affymetrix Porcine array is the most sensitive and reproducible microarray for swine genomic studies.

## Background

Gene expression profiling utilizing microarrays has become a widely used approach to elucidate biological function in complex systems. In mice and humans, a number of different platforms and approaches have been developed that have allowed gene expression analysis under a broad range of treatment conditions both *in vitro *and *in vivo*. In swine, in spite of limited genomic information available, several platforms have been developed for gene expression profiling. There are two microarrays currently available for porcine gene expression studies: Affymetrix Porcine (24,123 probe sets), and a U.S. Pig Genome Coordination Program glass spotted long oligonucleotide microarray (13,827 probes) [1,2]. In addition, a few groups have reported cross-species microarray hybridization onto Affymetrix Human arrays with mRNA from species such as dog, cattle, and swine [3,4], thus suggesting that the Affymetrix Human platform (54,676 probe sets) may also be useful in porcine gene expression studies. There has also been a single report of cross-species hybridization of porcine cDNA onto human nylon microarrays [5].

While Zhao *et al*. reports validation of the porcine glass spotted long oligonucleotide array [6], there are no reports thus far on the Affymetrix Porcine microarray released in early 2005. In order to determine which of the presently available microarrays would be preferable for swine gene-profiling studies we compared both porcine based platforms as well as the human Affymetrix arrays (in cross-species hybridization). There are compelling reasons why one might theoretically choose any of these three array platforms. The Affymetrix Human array has the greatest coverage and is well annotated against the human genome, but is complicated by the effects of cross-species hybridization. The Affymetrix Porcine array has an intermediate level of coverage but is poorly annotated. The glass spotted oligonucleotide array platform has the potential advantage of greater specificity and has a lower unit cost, but has the lowest coverage of all three platforms.

To ensure that we could assess platforms in terms of the biological relevance of the information generated, we chose to compare gene expression profiles of biparental and parthenogenetic porcine fibroblasts. Comparisons between control and parthenogenetic mouse embryos have been previously used to identify imprinted genes [7,8] and extensive information exists regarding expected differences in gene expression between these two cell populations. As such, results from the three platforms being compared can be examined not just for their technical reproducibility, but also for the relevance of the information expected. Specifically, diploid parthenogenetic embryos contain only maternally-derived chromosomes and as such they have two sets of maternally imprinted genes and no paternally imprinted genes. In contrast, normal biparental embryos contain one complement each of paternal and maternal imprinted genes. Comparison of the gene expression profiles of both groups allows the identification of imprinted genes as has been demonstrated by Mizuno *et al*. [8]. Thus, by using this model system it is possible not only to compare platforms for their technical merits but also for the extent of biological information generated (i.e. identification of known imprinted genes).

## Results

### Technical Reproducibility

**Figure 1:**
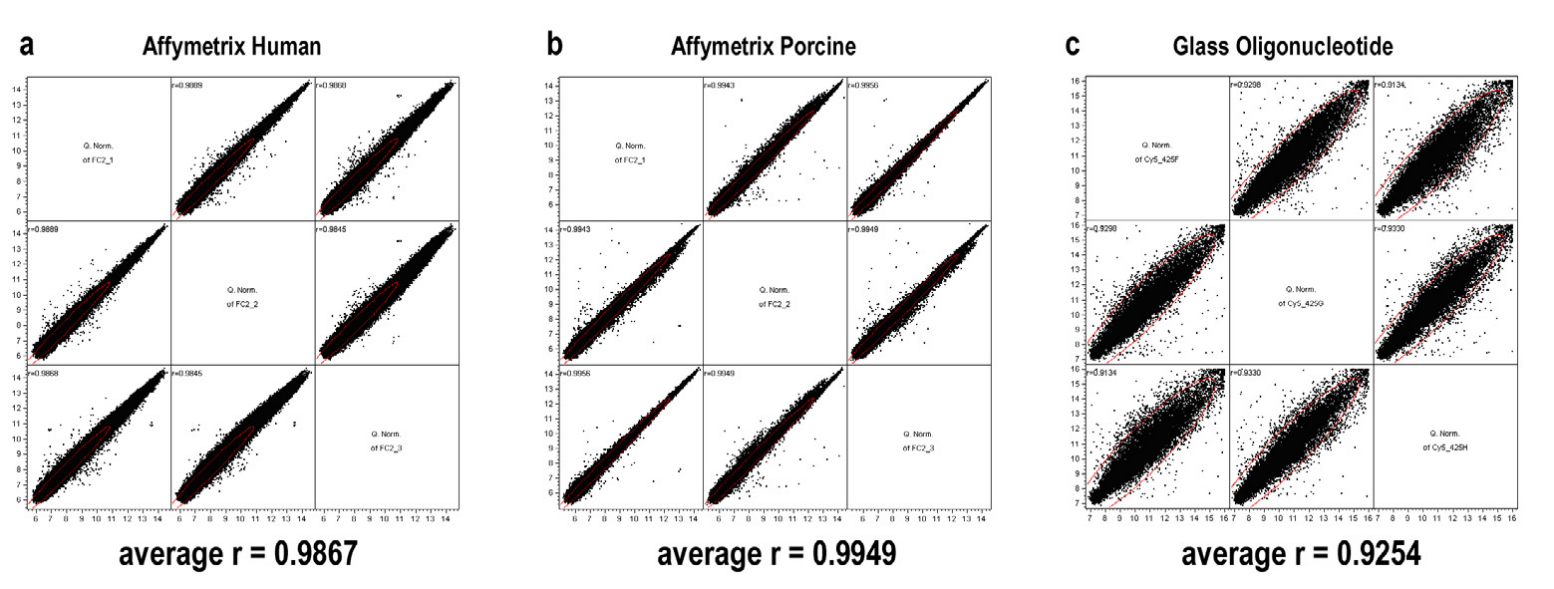
Reproducibility of technical replicates. Pairwise scatterplot of control technical replicates for (a) Affymetrix Human, (b) Affymetrix Porcine, and (c) long glass oligonucleotide array. Average Pearson correlation coefficients across three htechnical replicates are given.

All arrays were normalized (treatment of each array described in methods) and compared by pairwise correlations between technical replicates, with the average Pearson correlation coefficient given below. Affymetrix Human and Porcine arrays were both highly correlated between technical replicates indicating high technical reproducibility. The lower correlation between replicates of the Affymetrix Human array is likely due to the greater percentage of inherently more variable non-hybridizing probes caused by sequence divergence mismatches. Also, it was necessary to remove approximately 10% of the probes on the long glass oligonucleotide array due to printing defects prior to normalization [see Additional File 1]. After this procedure, it was found that although these spotted arrays performed relatively well, they nonetheless showed significantly more variability than the Affymetrix arrays. Decreased error variance due to high technical reproducibility is one of the key contributing factors to a platform's ability to identify differentially expressed genes (Figure 1).

### Cross-species hybridization onto Affymetrix Human Genechips

**Figure 2:**
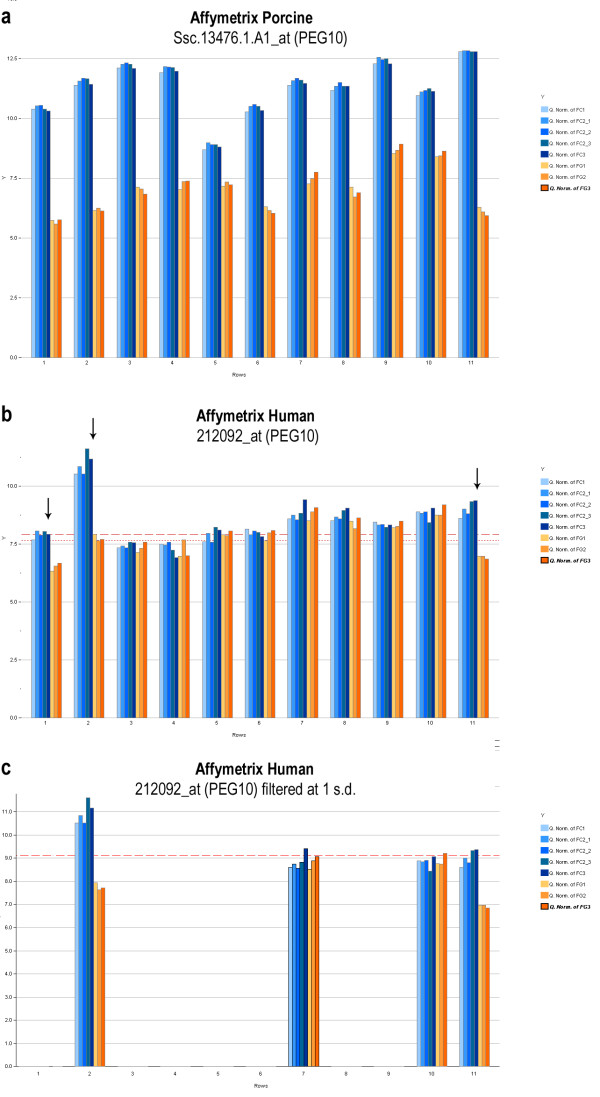
Effects of probe set filtering. (a) A well behaved probe set on the Affymetrix Porcine platform. Differential expression is evident across the entire probe set. (b) A typical probe set on the Affymetrix Human platform. Differential expression (black arrows) is evident in only on three of eleven probes. (c) Probe set filtering performed at a stringent threshold of one standard deviation above the mean array intensity (red dotted line). Note that while two of the probe sets exhibiting differential expression are captured, one is lost, and two probe sets which do not indicate differential expression also remain.

Utilizing the design described above, porcine cRNA was hybridized to human Affymetrix arrays and data analyzed by filtering and subsequent analysis via a linear mixed model as described in the methods. Due to the effects of cross-species hybridization, the complexity of downstream analysis for Affymetrix Human U133+2.0 arrays in the context of cross-species hybridization was significantly greater than for the remaining arrays. Specifically, a high degree of variability within probe sets, likely due to probes with low sequence identity between human and pig was noted. The plots of probe expression profiles highlights the difficulty of this problem (Figure 2a,2b).

In the Affymetrix Human probe set *212092_at*, targeting the gene *PEG10 *(Figure 2b), the small black arrows indicate that only 1^st^, 2^nd^, and 11^th ^probes appear to be differentially expressed in control versus parthenogenetic samples. The remaining probes show intensities that are randomly distributed around the median of the array. In contrast, *Ssc.13476.1.A1_at*, a porcine-specific probe for the same transcript (Figure 2a), showed clear evidence of differential expression across all probes. This inconsistent hybridization within probe sets is representative of the probes on the Affymetrix Human array under these cross-species hybridization conditions. Figure 2c further illustrates difficulties with the filtering process. In this figure, the dotted red line in the expression profile on the left is a filtering threshold. The probe set in the lower left shows results of filtering at a particular threshold (> one standard deviation from the mean intensity of the array). This illustrates how this type of filtering is imperfect as at a fairly stringent threshold, only two of three probe sets that exhibit evidence for differential expression are retained along with two probe sets that do not.

We tested a number of filtering thresholds based on the perfect match probe intensity in an effort to optimize this procedure (Table 1). However, far fewer differentially expressed known imprinted genes were identified by the Affymetrix Human array, even after applying these filtering procedures. A filtering procedure proposed by Ji. *et al*., which implements a filter based on the difference and ratio of perfect match and mismatch probes [4] was also tested. This filtering approach did not improve results in terms of known imprinted genes identified in comparison to filtering solely based on perfect match intensity (data not shown).

### Estimates of Differential Expression

**Figure 3:**
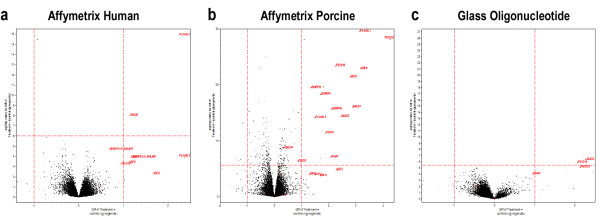
Volcano plots of differential expression. Volcano plots of estimate of differential expression for (a) Affymetrix Human, (b) Affymetrix Porcine, and (c) glass spotted long oligonucleotide microarray. These plots provide an immediate visual assessment of the degree of detectable differential gene expression as well as concordance with predicted differential gene expression.

Differential expression for all three platforms was determined by estimating treatment effects after fitting a linear mixed model using SAS and JMP/Genomics via the method of Wolfinger *et al*.. [9]. The volcano plots (plotting estimate of treatment effects against the negative log of the p-value) (Figure 3) demonstrate that the number of significant differentially expressed genes identified varies greatly between these three platforms. All known imprinted transcripts have been highlighted in red; the dotted red lines correspond to a Bonferroni adjusted (p < 0.05) on the vertical axis and > 2-fold change on the horizontal. The Affymetrix Porcine array identified both the greatest number of differentially expressed genes as well as the greatest number of differentially expressed known imprinted genes. This same trend was exhibited when the threshold for differential expression was set at q<0.05 and q<0.20. Table 2 summarizes the transcriptional differences identified.

### Sensitivity

**Figure 4:**
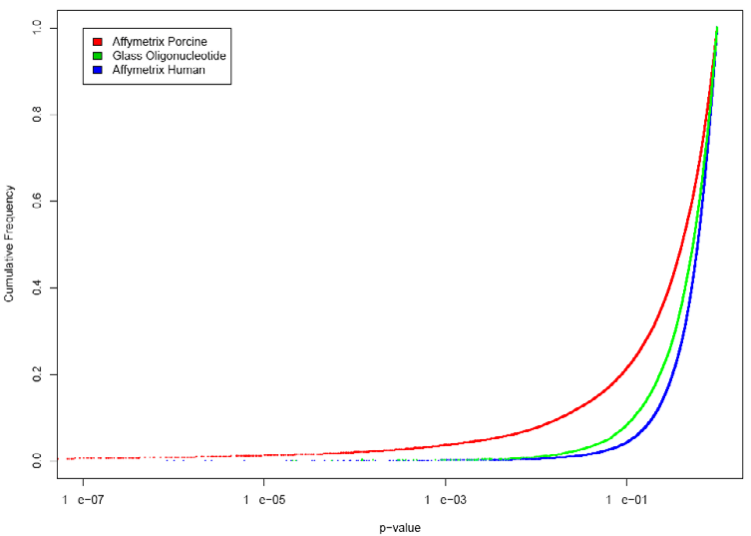
Cumulative distribution frequency of p-values. Cumulative distribution frequency of p-values for the three array platforms. This plot shows that at all p-values the highest number of differentially expressed genes is identified by the Affymetrix Porcine platform.

A cumulative distribution plot shows the proportion of genes that are at or below a full range of p-value thresholds (Figure 4). It corroborates that at all significance thresholds, the Affymetrix Porcine array detects genes as being differentially expressed with higher frequency.

### Identification of Sequence-Matched Probes

**Figure 5:**
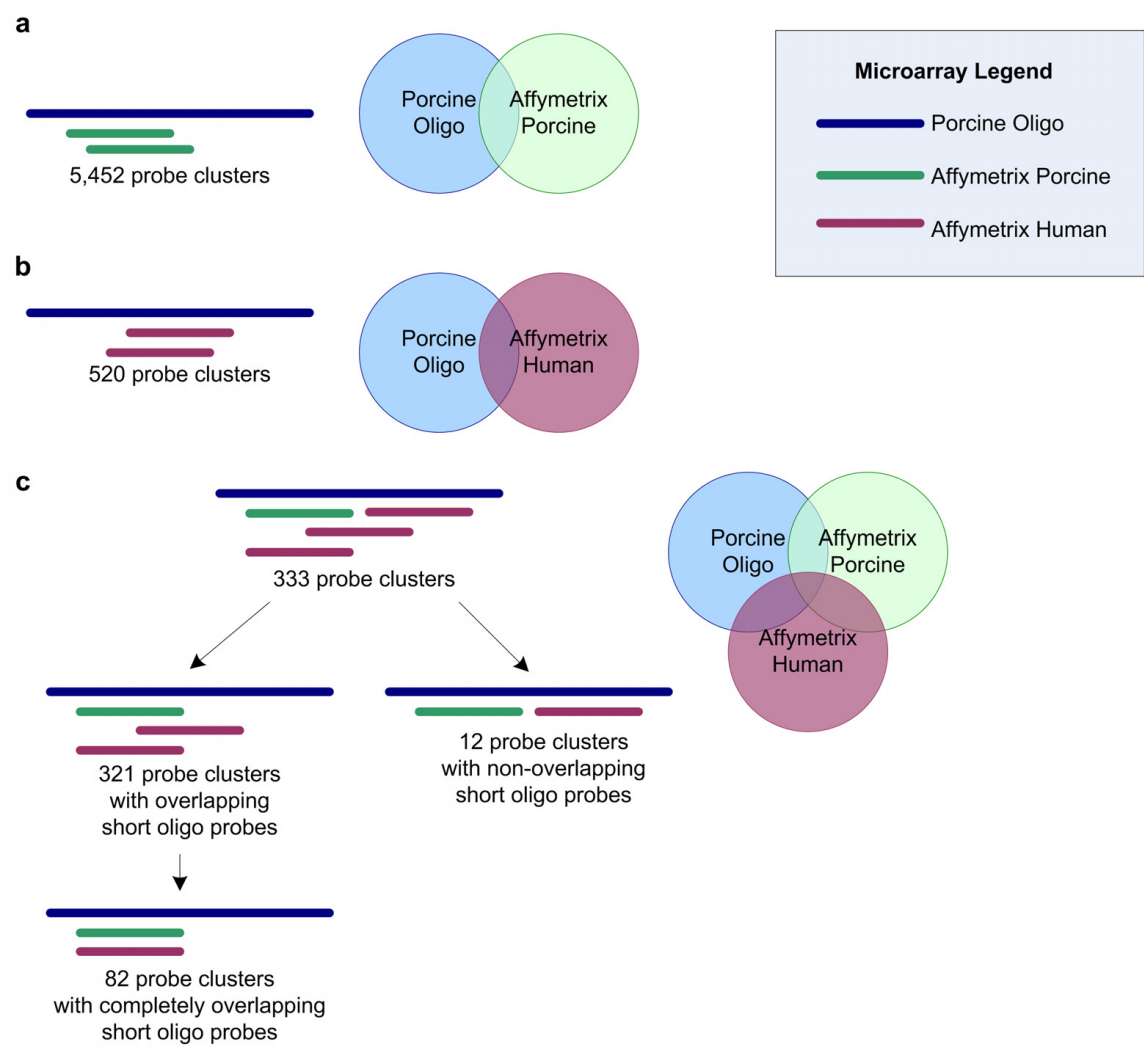
Summary of sequence-oriented probe matching. (a) 10,212 Affymetrix Porcine short oligonucleotides map to Porcine Glass long oligonucleotides. They cluster into 5,452 probe clusters around each long oligonucleotide sequence. (b) 727 Affymetrix Human short oligonucleotides map to Porcine Glass long oligonucleotides, clustering into 520 probe clusters. (c) The intersection of these two mappings results in 333 probe clusters which are used to assess intra-platform reproducibility. 82 of these probe clusters contain short oligonucleotide sequences that match exactly between the two Affymetrix platforms.

In order to assess the performance of the microarrays independently of coverage, we identified a set of 333 probe clusters common to all platforms by sequence-based mapping on the probe level. Briefly, we selected short oligonucleotide probes that mapped to long oligonucleotide probes with complete sequence identity and included them in a matching probe cluster where there were mappings for both short oligonucleotide Affymetrix microarrays to the single porcine glass long oligonucleotide microarray. 10,212 Affymetrix Porcine short oligonucleotides mapped to 5,452 unique Porcine Glass long oligonucleotides; 727 Affymetrix Human short oligonucleotides map to 520 unique Porcine Glass long oligonucleotides. The intersection of these two mappings results in the 333 probe clusters which are used to assess intra-platform reproducibility. 82 of these probe clusters contain short oligonucleotide sequences that match exactly between the two Affymetrix platforms; correlation coefficients are also calculated for this subset. (Figure 5)

Within the constraints of the technology (70-mer probes versus 25-mer probes), this sequence-based probe-to-probe mapping is the most rigorous method of identifying comparable matching probes to assess inter-platform reproducibility.

### Inter/Intra-Platform Reproducibility

Average pairwise Pearson and Spearman correlation coefficients were calculated, showing strong correlation both within and between the two Affymetrix microarrays, but moderate correlation between the porcine glass and the Affymetrix platforms. As expected, given the high strong intra-platform correlation of the Affymetrix microarrays, probes with identical sequence between Affymetrix Porcine and Affymetrix Human microarrays also show very strong correlation (Table 3).

These correlation values compare favorably with the best correlations reported by Pylatuik *et al*. on biological replicates between platforms and are comparable to the correlations for *A *values obtained by Barczak *et al*. where technical replicates were used to compare the same sample between short and long oligonucleotide platforms (Table 3; [10,11]).

### Validation of known imprinted genes by qRT-PCR

Validation of microarray results by real-time quantitative reverse-transcription PCR (qRT-PCR) was conducted by examining expression of known imprinted transcripts. A subset of known imprinted transcripts were selected regardless of their levels of expression on the various microarrays. Our results (Table 4) indicate that for those transcripts where the individual microarrays showed significant evidence for differential expression (*PEG10, PLAGL1*, *SGCE*, and *IGF2) *there was qualitative agreement with the results of qRT-PCR. At a more relaxed threshold, SGCE would also be detected as significantly differentially expressed on the glass oligonucleotide array, while PLAGL1 and IGF2 are not represented on the glass arrays. There were two transcripts, DLX5 and DCN, which were detected as differentially expressed by qPCR but not by the microarrays at a statistically conservative Bonferroni corrected p < 0.05 cutoff. This is consistent with the observation that microarrays tend to underestimate absolute fold change and that qRT-PCR has greater detection sensitivity (but considerably lower throughput) than microarray technology [12].

## Discussion

The difficulty with assessing relative performance of microarrays is that the truth regarding which genes are actually differentially expressed is not known. It is not feasible in practice to validate all but a relatively small sampling of genes by quantitative PCR, thus, surrogate measures for accuracy such as concordance are typically used in comparisons between platforms. In this study, we've used several classical metrics for reproducibility and sensitivity of detection. In addition, we've chosen to use the number of known imprinted genes identified as an *ad hoc *measure of the performance of each platform under realistic experimental conditions. We find that Affymetrix Porcine arrays were the most technically reproducible and were able to identify the greatest number (and highest percentage) of currently known imprinted genes, while Affymetrix Human and Porcine Glass Oligonucleotide arrays identify a comparable number (Table 2). The biological significance of this study is that it suggests that the following genes: DIRAS3, MEST, NDN, NNAT, SGCE, SRNPN, PEG3, PLAGL1, and PEG10 are imprinted in porcine fibroblast. Their prominent disregulation may point to a role in the failure of parthenogenetic development to term in swine.

It has been suggested that it might be possible to run additional glass long oligonucleotide arrays to compensate for their increased technical variability, particularly as they have a lower unit cost. While a larger number of arrays may be able to compensate and provide increased statistical power to a glass long oligonucleotide array experiment, it is not relevant to the remaining issue of considerably lower coverage.

The primary complication with cross-species hybridization of porcine derived cRNA onto Affymetrix Human Genechips is the difficulty in differentiating whether observed low signal intensity is due to low transcript abundance or sequence divergence. Without genome sequence to resolve this question, the utility of these arrays for porcine gene expression studies is reduced. Furthermore, though filtering at varying thresholds does appear to improve the detection of differentially expressed known imprinted genes there is not a clear optimum, and varying sets of known imprinted genes are detected at different thresholds. The optimal threshold may vary with sample condition and may thus be difficult to determine without known differentially expressed genes as controls, as we have in this case. This would pose an obstacle to typical experiments without *a priori *information about differential expression. We propose that while it is possible to identify differentially expressed genes by this method, with the availability of the Affymetrix Porcine array it is no longer necessary to perform this cross-species hybridization procedure in swine unless one is specifically interested in questions such as the degree of interspecies sequence similarity [13]. Overall, our observation that is that the effective coverage of Affymetrix Human arrays for detecting differential expression (using both PM-only and PM and MM based filtering methods) is less than that of Affymetrix Porcine arrays.

In other species where a commercial short oligonucleotide array is not available, the only available option may be cross-species hybridization onto a similar Affymetrix array. We observed that probe level filtering based only on perfect match probe intensities performs comparably to the perfect match and mismatch based approach of Ji *et al*. One possible reason that these two filtering procedures perform similarly despite fairly different implementations is because the Ji *et al*. procedure uses the Affymetrix MAS5 algorithm to obtain summary expression intensities. Since MAS5 is an expression summary based on the difference in intensity between perfect match and mismatch probes, it is logical and consistent to use a filtering scheme also based on this difference. We obviate the need to consider the mismatch probes when filtering by estimating differential expression using an approach based on perfect match intensities only and using this approach we obtain satisfactory and comparable results to a PM-MM filtering scheme which we implemented as described in Ji *et al*. [4].

There are open questions about whether data from different microarrays is comparable given low levels of concordance observed between different microarray platforms [14,15]. Spotted long oligonucleotide platforms are considered to be an improvement over cDNA arrays as it eliminates the problem of clone misidentification, although incorrect spot placement is still possible [16]. Our observation of moderate correlations between platforms is consistent with earlier studies. It reinforces the idea that, particularly in the absence of known reference standards, microarrays are better suited for identifying relative as opposed to absolute quantitative differences. The results of our microarray analyses, taken together with results of real-time quantitative PCR, suggest that microarrays are generally successful at identifying differentially expressed genes and identifying the same biological group of imprinted genes that were predicted to be differentially expressed *a priori *based on the parthenogenetic animal model along with empirical evidence from prior studies.

## Conclusion

In summary, results presented here indicate that the Affymetrix Porcine arrays have higher sensitivity and technical reproducibility in comparison to a porcine long glass oligonucleotide platform and cross species hybridization onto an Affymetrix Human platform. In addition, we have expanded the utility of these porcine microarrays through development of a more comprehensive annotation [17]. This enhanced annotation increases the amount of biological information that can be derived from the Affymetrix Porcine microarrays and increases their usefulness for swine genomic studies.

## Methods

### Experimental Design

**Figure 6:**
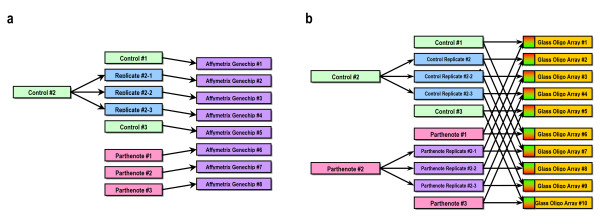
Experimental design. (a) Experimental design for Affymetrix arrays, with three biological replicates for each of two treatments (control and parthenote). One biparental control biological replicate is further split into a total of three technical replicates. (b) Experimental design for glass long oligonucleotide array with three biological replicates of each of two treatments (control and parthenote). One biparental control biological replicate and one parthenogenetic biological replicate are each split into three technical replicates.

Gene expression profiles of fibroblast cell lines derived from day 27 control and parthenogenetic embryos were compared in each of the three platforms. For each platform, three biological replicates were used. Each biological replicate consisted of fibroblasts derived from a randomly selected fetus and cultured for two passages. One of the biological replicate was further split into three technical replicates. For biparental controls, sex of fetuses was determined by PCR and only female fetuses were used to avoid sex-related gene expression differences. For the technical replicates, one of the biological replicates was split into three identical pools of RNA and hybridized independently. For cross-platform comparisons, the same starting pool of total RNA was used to generate labeled targets for each of the three individual experiments. A balanced dye swap design was employed for the two-channel glass oligonucleotide microarray and one control and one parthenogenetic biological replicate were each divided into three technical replicates (Figure 6).

### Generation of control pregnancies

Control crossbred gilts were mated by artificial insemination with boars to produce the biparental control fetuses for this study. Gilts were mated at 12 and 24 hr after their natural standing heat.

### Generation of parthenogenetic pregnancies

Oocyte collection and maturation: Porcine ovaries were collected from sows at a local slaughterhouse and transported in 0.9% saline solution at 30–35°C. At the lab, ovaries were washed four times with warmed saline solution. Cumulus oocyte complexes (COCs) were aspirated from ovarian follicles 3–8 mm in diameter using a 5 ml syringe fitted with a short bevel 18-gauge needle. Follicular fluid was collected in 50 ml centrifuge conical tubes at room temperature. Collected COCs were washed three times in TLH-PVA medium. Oocytes with uniform cytoplasm and at least two layers of compacted cumulus cells were used for maturation. COCs were matured in TC199-Hepes supplemented with 10% porcine follicular fluid (pFF), 5 μg/ml insulin, 10 ng/ml EGF, 0.6 mM cysteine, 0.2 mM pyruvate, 25 μg/ml kanamycin and 5 μg/ml of each eCG and hCG. Fifty COCs were cultured in 500 μl medium in a 4-well Nunc dish at 38.5°C, 5% CO_2 _in a humidified atmosphere. COCs were cultured for 22 hr before being changed to the same but eCG- and hCG-free culture medium and cultured for additional 15 hr [18].

### Electrical activation of pig oocytes

After 40 hr of maturation *in vitro*, cumulus cells of IVM pig oocytes were removed by repeated pipetting in 0.1% hyaluronidase. Denuded oocytes were washed three times in Ca^2+ ^free-NCSU23 medium [19] and then exposed for 5 min to activation medium consisting of 0.3 M mannitol, 0.05 mM MgSO_4_, and 0.1 mM CaCl_2_. Oocytes were then transferred between electrodes (1 mm apart) covered by 3 ml of the activation medium in a chamber connected to an electrical pulsing machine (BTX ECM 2001). Oocytes were stimulated by a single DC pulse of 150 V/mm for 100 μsecs. After activation, oocytes were washed twice in NCSU-23 medium supplemented with 0.4% BSA (IVC medium) and moved into IVC medium containing 10 μg/ml of cyclohexamide and incubated for 6 hr in this medium. Then, oocytes were washed three times in NCSU-23 medium supplemented with 0.05% BSA for transfer.

### Embryo transfer into recipient

Activated oocytes were transferred into naturally cycling gilts on the first day of the standing estrous. Ventral laparotomy was performed and oocytes were transferred into the oviduct [18].

### Collection of biparental and parthenogenetic fetuses

Pregnancies were confirmed by ultrasound two days before the collection on day 27. Fetuses were collected following euthanasia of the gilt and dissection of the reproductive tract. Fetuses were removed from their placenta, weighed and placed into 50 ml conical tubes containing DMEM supplemented with 10% fetal bovine serum (FBS). Tubes were kept on ice for transportation to the laboratory. Placentas were taken separately, weighed and placed in liquid nitrogen for later studies.

### Isolation of fibroblasts from biparental and parthenogenetic fetuses

The head and viscera of fetuses were removed and the remaining tissue was minced with a sterile razor blade. The tissue was added to 10 ml of 0.05% trypsin (Gibco) supplemented with 0.9 mM potassium chloride, 0.9 mM dextrose, 0.7 mM sodium bicarbonate, 0.1 mM EDTA (all from Sigma), and 20 mM sodium chloride (EMD Bioscience). The tissue/trypsin solution was shaken at 37°C for 15 min a total of three times. After incubation, the supernatant was collected, pooled, and pelletted. The cell pellet was resuspended in DMEM/F12 media (Gibco) supplemented with 10% FBS and 5% calf serum (CS) (both from Hyclone), 30 mM sodium bicarbonate, 0.5 mM pyruvic acid, and 2 mM N-acetyl-L-cysteine (all from Sigma). In addition, 100 units penicillin and 100 ug streptomycin,(Gibco) were added per 100 ml media to inhibit microbial growth. The cells were placed in the appropriate number of 10 cm tissue culture plates (Corning), incubated in a 5% CO_2 _incubator at 37°C, expanded once and frozen in 50% FBS, 40% media, and 10% DMSO (Sigma) for long time storage and future use.

### Determination of sex of fetuses by PCR

The sex of the fetuses from which each of the biparental control fibroblast cell lines was derived was determined by SRY genotyping using the following primers: 5'-TGAACGCTTTCATTGTGTGGTC-3', 5'-TCCTCCGTGTCTCTGATGACCG-3' [20]. The PCR thermocycling conditions were 95°C for 2 min, 35 cycles of 95°C for 20 s, 55°C for 30 s, 72°C for 1 min, followed by 72°C for 7 min.

### RNA Isolation

Cells derived from biparental female and parthogenetic fetuses were grown in 10 cm tissue culture plates in DMEM/F12 media (Gibco) supplemented with 10% FBS and 5% calf serum CS (both from Hyclone), 30 mM sodium bicarbonate, 0.5 mM pyruvic acid, and 2 mM N-acetyl-L-cysteine (all from Sigma). At 90% confluency, the RNA was extracted using RNAqueous Kit (Ambion) as per the instructions of the manufacturer. Briefly, media was removed from the plates and cells were lysed in 1 ml lysis buffer. To this was added 1 ml of 64% alcohol. The contents were mixed and passed through the column. The RNA bound to the column was washed once with wash solution 1 and twice with wash solution 2/3. Finally RNA was eluted in 40 *μ*l of hot elution buffer. RNA was quantified by spectrophotometry and quality verified by running 5 *μ*g of RNA on 1% agarose gel. The resulting RNA was used for microarray analyses.

### Target Production and Hybridization: Affymetrix Human and Porcine arrays

Before target production, the quality and quantity of each RNA sample was assessed using a 2100 BioAnalyzer (Agilent). Target was prepared and hybridized according to the Affymetrix Technical Manual. Total RNA (10 ug) was converted into cDNA using Reverse Transcriptase (Invitrogen) and a modified oligo(dT)_24 _primer that contains T7 promoter sequences (GenSet). After first strand synthesis, residual RNA was degraded by the addition of RNaseH and a double-stranded cDNA molecule was generated using DNA Polymerase I and DNA Ligase. The cDNA was then purified and concentrated using phenol:chloroform extraction followed by ethanol precipitation. The cDNA products were incubated with T7 RNA Polymerase and biotinylated ribonucleotides using an In Vitro Transcription kit (Enzo Diagnostics). One-half of the cRNA product was purified using an RNeasy column (Qiagen) and quantified with a spectrophotometer. The cRNA target (20 ug) was incubated at 94°C for 35 min in fragmentation buffer (Tris, Magnesium Acetate, Potassium Acetate). The fragmented cRNA was diluted in hybridization buffer (MES, NaCl, EDTA, Tween 20, Herring Sperm DNA, Acetylated BSA) containing biotin-labeled OligoB2 and Eukaryotic Hybridization Controls (Affymetrix). The hybridization cocktail was denatured at 99°C for 5 min, incubated at 45°C for 5 min and then injected into a GeneChip cartridge. The GeneChip array was incubated at 42°C for at least 16 hr in a rotating oven at 60 rpm. GeneChips were washed with a series of nonstringent (25°C) and stringent (50°C) solutions containing variable amounts of MES, Tween20 and SSPE. The microarrays were then stained with Streptavidin Phycoerythrin and the fluorescent signal was amplified using a biotinylated antibody solution. Fluorescent images were detected in a GeneChip^® ^Scanner 3000 and expression data were extracted using the MicroArray Suite 5.0 software (Affymetrix). All GeneChips were scaled to a median intensity setting of 500.

### Target Production and Hybridization: U.S. Pig Genome Coordination Program Glass Arrays

RNA was extracted from primary cultures of control and gynogenote fibroblasts using RNAqueous^® ^(Ambion) following the manufacturer's suggested protocol and stored at -80°C. One microgram of purified RNA was converted to aminoallyl-coupled RNA (aRNA) and coupled with Cy3 or Cy5 using Amino Allyl Message Amp II aRNA Kit (Ambion), again suggested protocols were followed. Specific activity and aRNA concentration of the purified labeled aRNA was determined by assaying one μl of sample on a NanoDrop^® ^ND 1000. Specific activities (pmol dye/pmol aRNA) of all probes were between 25 and 40. Control and parthenogenetic probes were pooled so that equal molar amounts of each dye were used per array. Pooled probes were dried to completion using an Eppendorf Vacufuge then fragmented using Fragmentation Reagent (Ambion). Fragmented probes were dried to a 10 μl volume and immediately used for hybridization. Glass arrays were generated at the University of Minnesota Microarray Printing Facility and obtained through the U.S. Pig Genome Coordination Program. Arrays were used within two weeks of receipt. Slides were pre-hybridized, hybridized and washed according to GAPS II Coated Slides instruction manual (Corning) with the exception that 300–400 picomoles of dye were used per slide and 0.1 μg/μl of Thymus DNA (Sigma) was used in the hybridization buffer. The arrays were scanned with ScanArray Express (Packard Bioscience). Acquired images were analyzed using QuantArray software version 3.0 (Packard Bioscience). During the quantification process, approximately 10% of probes were discarded due to visually identified printing defects on the arrays.

### Normalization and Filtering

For Affymetrix arrays, probe intensity values were log2 transformed and quantile normalization was applied [21]. The average of the three technical replicates was taken to determine the probe intensities for the corresponding biological replicate. The Affymetrix Human arrays were treated as a special case due to the effects of sequence divergence on cross-species hybridization. For these arrays, we employed quantile normalization, and then subsequently corrected for the increased variability of probe expression profiles by filtering out non-hybridizing probes from within probe sets. We tried two approaches to filtering, one solely based on the intensity of the perfect match probe, and the second based on both the difference and the ratio between the perfect match and the mismatch probe as described by Ji et al. [4]. In the perfect match only approach, we filtered out non-hybridizing probes which did not exceed arbitrary filtering thresholds in any of the samples. We tried four filtering thresholds: 0, the median array intensity, the third quartile, and one standard deviation above the mean. In the perfect match and mismatch approach, we implemented a filter at PM - MM > 200, PM/MM > 2.

The spotted glass oligonucleotide arrays were normalized with a lowess normalization with a smoothing parameter of 0.2 to broadly correct for dye effects.

### Statistical Analysis

The Affymetrix arrays were fitted to the following gene by gene linear mixed model using SAS and JMP/Genomics (Cary, NC) [9].

*y*_*ijk *_= μ + *T*_*i *_+ *P*_*j *_+ *A*_*k *_+ *ε*_*ijkl*_

For each probe set, *y *is the log2 transformed intensity of the *i*^th ^treatment, j^th ^probe, and k^th ^array. This model included fixed effects for treatment (control or parthenote, *T*) and probe (*P) *and random effects for array (*A*).

The glass spotted oligonucleotide array was fitted to the following gene by gene linear mixed model.

*y*_*ijk *_= μ + *T*_*i *_+ *D*_*j *_+ *A*_*k *_+ *D*_j_*A_*k *_+ *ε*_*ijkl*_

For each probe, *y *is the log2 transformed intensity of the *i*^th ^treatment, j^th ^dye, and k^th ^array. This model included fixed effects for treatment (control or parthenote, *T*), dye (*D)*, the interaction between dye and treatment (D*A), and random effects for the array (*A*).

Least square means were estimated for the difference between treatments for each gene. In the Affymetrix Human array, corresponding p-values were converted to q-values by a method proposed by Storey that measures significance in terms of false discovery rate to optimize filtering thresholds [22]. For all arrays, p-values were adjusted with a Bonferroni correction to control the family wide error rate to <0.05.

### Correlations for technical reproducibility

The control technical replicates for each of the three array platforms were compared by standard pairwise correlations. The average Pearson correlation coefficient for these three arrays is reported. The Cy5 channel of the glass array is used for comparison purposes, but both channels have similar correlation values.

### Cumulative distribution of p-values

An empirical cumulative distribution function was fitted for each of the three sets of p-values. A plot of p-value by frequency was then constructed, where each point represents a gene with its corresponding p-value.

### Identification of Sequence-Matched Probe Clusters

Mecham *et al*. have suggested that the lack of concordance in cross-platform microarray comparisons may be caused by a reliance on gene annotations without more stringent sequence-oriented matching of probes [23]. We downloaded probe sequences represented on the microarrays from Affymetrix [24,25], and Operon [26]. Using this sequence information, probe sequences were matched at the probe level by mapping Affymetrix short oligonucleotides to porcine spotted glass long oligonucleotide sequences using the BLAST standalone program as described by Kuo *et al*. [27]. Using the long oligonucleotide sequences as a reference, probe clusters were identified where there is a match with complete sequence identity between the long oligonucleotide sequences and short oligonucleotide sequences for the span of the short oligonucleotide sequence for both Affymetrix microarrays (matching probe clusters). In the cases where there was more than one short oligo probe per microarray per cluster, the average of the normalized expression intensities was taken. A subset of matching probes with complete overlap were identified where the short oligonucleotide sequences on the two Affymetrix microarrays have identical sequence.

### Intra/Inter-platform correlations

Average Pearson and Spearman pairwise correlation coefficients were calculated on the normalized expression intensities of the control technical replicates using JMP (Cary, NC). Additionally, Pearson and Spearman pairwise correlation coefficients were calculated for the subset of matching probes with complete overlap.

### Annotation

Since the two porcine microarrays were minimally annotated, both were reannotated by BLAST against an EnsEMBL Human cDNA sequence library. This annotation was enhanced by using information from The Institute for Genome Research (TIGR) Pig Gene Index [17]. Briefly, we attempted to extend the target sequences by matching them to TIGR assembled porcine consensus sequences. These extended target sequences were compared to a library of EnsEMBL human cDNA sequences by BLAST, and the gene with the highest bit score was recorded. The same procedure was repeated for the original unextended target sequences. A subset of these original sequences with bit scores greater than 50 were evaluated for concordance with the extended target sequences and resulted in >96% agreement.

### Validation by qRT-PCR

Gene transcripts were quantified by real-time reverse transcription PCR using the iCycler apparatus (BioRad Inc., Hercules, CA) and were detected with SYBR Green I as fluorochrome (Platinum SYBR Green I; Invitrogen, Carlsbad, CA). The primers used for PCR are listed in Additional File 2. The relative expression changes were determined with the 2−ΔΔCt
 MathType@MTEF@5@5@+=feaafiart1ev1aaatCvAUfKttLearuWrP9MDH5MBPbIqV92AaeXatLxBI9gBaebbnrfifHhDYfgasaacH8akY=wiFfYdH8Gipec8Eeeu0xXdbba9frFj0=OqFfea0dXdd9vqai=hGuQ8kuc9pgc9s8qqaq=dirpe0xb9q8qiLsFr0=vr0=vr0dc8meaabaqaciaacaGaaeqabaqabeGadaaakeaacqaIYaGmdaahaaWcbeqaaiabgkHiTiabfs5aejabfs5aejabboeadnaaBaaameaacqqG0baDaeqaaaaaaaa@342D@ method, where ΔCt=Cttarget gene internal reference
 MathType@MTEF@5@5@+=feaafiart1ev1aaatCvAUfKttLearuWrP9MDH5MBPbIqV92AaeXatLxBI9gBaebbnrfifHhDYfgasaacH8akY=wiFfYdH8Gipec8Eeeu0xXdbba9frFj0=OqFfea0dXdd9vqai=hGuQ8kuc9pgc9s8qqaq=dirpe0xb9q8qiLsFr0=vr0=vr0dc8meaabaqaciaacaGaaeqabaqabeGadaaakeaacqqHuoarcqqGdbWqdaWgaaWcbaGaeeiDaqhabeaakiabg2da9iabboeadnaaBaaaleaacqqG0baDdaWgaaadbaGaeeiDaqNaeeyyaeMaeeOCaiNaee4zaCMaeeyzauMaeeiDaqNaeeiiaaIaee4zaCMaeeyzauMaeeOBa4MaeeyzauMaeeiiaaIaeeyAaKMaeeOBa4MaeeiDaqNaeeyzauMaeeOCaiNaeeOBa4MaeeyyaeMaeeiBaWMaeeiiaaIaeeOCaiNaeeyzauMaeeOzayMaeeyzauMaeeOCaiNaeeyzauMaeeOBa4Maee4yamMaeeyzaugabeaaaSqabaaaaa@5BA0@, and ΔΔCt=ΔCtsample−ΔCtcalibrator
 MathType@MTEF@5@5@+=feaafiart1ev1aaatCvAUfKttLearuWrP9MDH5MBPbIqV92AaeXatLxBI9gBaebbnrfifHhDYfgasaacH8akY=wiFfYdH8Gipec8Eeeu0xXdbba9frFj0=OqFfea0dXdd9vqai=hGuQ8kuc9pgc9s8qqaq=dirpe0xb9q8qiLsFr0=vr0=vr0dc8meaabaqaciaacaGaaeqabaqabeGadaaakeaacqqHuoarcqqHuoarcqqGdbWqdaWgaaWcbaGaeeiDaqhabeaakiabg2da9iabfs5aejabboeadnaaBaaaleaacqqG0baDdaWgaaadbaGaee4CamNaeeyyaeMaeeyBa0MaeeiCaaNaeeiBaWMaeeyzaugabeaaaSqabaGccqGHsislcqqHuoarcqqGdbWqdaWgaaWcbaGaeeiDaq3aaSbaaWqaaiabbogaJjabbggaHjabbYgaSjabbMgaPjabbkgaIjabbkhaYjabbggaHjabbsha0jabb+gaVjabbkhaYbqabaaaleqaaaaa@526D@. 18S was used as internal reference gene. PCR efficiency was tested for each primer pair by 10-fold dilution series of cDNA in triplicate to make sure that efficiency is appropriate for the 2^-ΔΔCt ^Pfaffl *et al*. method [28]. To ensure the specificity and integrity of the PCR product, melt-curve analyses were performed for all PCR products. No PCR products were obtained from RNA samples when RT was omitted. Samples without template for each primer pairs were included to identify contamination. The experimental design was executed in triplicate for each control and parthenote combination.

## Authors' contributions

ST performed the microarray analyses and drafted the manuscript. JE generated and collected the control and parthenogenetic embryos. BM established cell lines and isolated total RNA. AM performed the glass microarray hybridizations. SB performed microarray validation by qPCR. JP is the principal investigator of the laboratory, conceived of the study, and participated in its design and coordination. All authors read and approved the final manuscript.

## Supplementary Material

Additional file 1 Example of printing defect in glass oligonucleotide arrays. Example of printing defects associated with glass long oligonucleotide arrays juxtaposed with well defined spots from one region of the microarray. These defects are characteristic of spot overprinting and have a clear impact on array to array variability. Spots that are obviously defective were removed prior to array quantitation. Click here for file

Additional file 2 Primers used in qRT-PCR validation of microarray data. These are the primer sequences used in qPCR validation of the microarray data. Click here for file

## Figures and Tables

**Table 1:** As the threshold stringency increases, the number of known imprinted genes identified as differentially expressed at a q < 0.05 reaches a local optimum while the number of significant genes decreases.

**Effects of filtering at various thresholds on the Affymetrix Human array**								
**Filter Thresholds**	**PM Threshold**	**Number of Probes After Filtering**	**% Probes After Filtering**	**% Probe Sets Remaining**	**Number Significant (q < 0.05)**	**Number Known Imprinted (q < 0.05)**	**Number Significant (p**_**adj**_**< 0.05)**	**Number Known Imprinted (p**_**adj**_**< 0.05)**

No filter	0.000	604,258	100.0%	100.00%	21	4	8	2
Median	7.655	361,110	59.8%	99.11%	20	6	7	2
75% quartile	8.479	197,484	32.7%	92.80%	14	6	4	2
1 standard deviation above mean	9.104	113,455	18.8%	79.20%	6	3	3	2

**Table 2:** The number of DEGs is determined at a Bonferroni-adjusted significance level of 0.05 for each of the three microarrays.

**Statistics on coverage and detection of differentially expressed genes (DEGs)**						
Platform	Total Number of Genes	Number of DEGs	Number of DEGs > 2-fold change	Known Imprinted Genes Represented	Number of KnownImprinted Genes Differentially Expressed	% Known Imprinted Genes Differentially Expressed

Affymetrix Human (filtered, 75% quartile)	50,737	4	2	215	2	0.9
Affymetrix Porcine	24,123	210	19	111	13	11.7
Glass Oligonucleotide	13,827	3	2	29	1	3.4

**Table 3:** Average Pearson and Spearman correlations were calculated between normalized intensities for control technical replicates. All correlations are significant at p < 0.0001. These results show strong correlation both within and between the two Affymetrix microarrays, but lower correlation between the porcine glass and the Affymetrix platform.

**Sequence-oriented Inter/Intra-Platform Correlation**						
	Correlation Coefficients of Matching Probes					
	
	Affymetrix Human		Affymetrix Porcine		Porcine Glass	
			
	Pearson	Spearman	Pearson	Spearman	Pearson	Spearman
Affymetrix Human	0.983	0.985	0.842	0.837	0.555	0.560
Affymetrix Porcine	0.842	0.837	0.998	0.998	0.618	0.624
Porcine Glass	0.555	0.560	0.618	0.624	0.837	0.827
	Correlation Coefficients of Matching Probes with Complete Overlap					
	
	Affymetrix Human		Affymetrix Porcine		Porcine Glass	
			
	Pearson	Spearman	Pearson	Spearman	Pearson	Spearman

Affymetrix Human	0.987	0.979	0.980	0.970	0.628	0.588
Affymetrix Porcine	0.980	0.970	0.997	0.996	0.633	0.620
Porcine Glass	0.628	0.588	0.633	0.620	0.844	0.827

**Table 4:** Results of validation by qRT-PCR alongside detection of differential expression by microarray demonstrating qualitative agreement.

**qPCR Validation**								
**Gene Name**	**2**^ΔΔCT^	**2**^ΔΔCT ^standard deviation	**qPCR p-value**	**qPCR**	**Direction of Imprinted Expression**	**Porcine Spotted Long Oligo**	**Human Short Oligo**	**Porcine Short Oligo**
*PEG10*	785544.42	9.3	<0.0001	Yes	Paternal	Yes	Yes	Yes
*PLAGL1*	758.32	1.4	<0.0001	Yes	Paternal	No	Yes	Yes
*SGCE*	450.90	2.1	<0.0001	Yes	Paternal	No	Yes	Yes
*IGF2*	24.53	1.9	0.0010	Yes	Paternal	No	Yes	Yes
*DLX5*	7.53	1.5	0.0065	Yes	Maternal	No	No	No
*DCN*	5.04	1.6	0.0168	Yes	Maternal	No	No	No
*GRB10*	1.38	2.2	0.1913	No	Maternal	No	No	No
*CD81*	0.98	1.6	0.2026	No	Maternal	No	No	No
*COMMD1*	0.86	2.8	0.3265	No	Maternal	No	No	No
*CDKN1C*	0.79	6.7	0.4283	No	Maternal	No	No	No
*IGF2R*	0.74	1.3	0.2119	No	Maternal	No	No	No
