# Supplementary material for: Detection of transcriptional difference of porcine imprinted genes using different microarray platforms
Source: BMC Genomics. 2006 Dec 28;7:328. doi: 10.1186/1471-2164-7-328 (PMC1769376; doi:10.1186/1471-2164-7-328)
Supplement: Additional file 1 — Example of printing defect in glass oligonucleotide arrays. Example of printing defects associated with glass long oligonucleotide arrays juxtaposed with well defined spots from one region of the microarray. These defects are characteristic of spot overprinting and have a clear impact on array to array variability. Spots that are obviously defective were removed prior to array quantitation. [file 1471-2164-7-328-S1.doc]

**Additional File 1.** Example of printing defects associated with glass long oligonucleotide arrays juxtaposed with well defined spots from one region of the microarray. These defects are characteristic of spot overprinting and have a clear impact on array to array variability. Spots that are obviously defective were removed prior to array quantitation.
