# Supplementary material for: Detection of transcriptional difference of porcine imprinted genes using different microarray platforms
Source: BMC Genomics. 2006 Dec 28;7:328. doi: 10.1186/1471-2164-7-328 (PMC1769376; doi:10.1186/1471-2164-7-328)
Supplement: Additional file 2 — Primers used in qRT-PCR validation of microarray data. These are the primer sequences used in qPCR validation of the microarray data. [file 1471-2164-7-328-S2.doc]

**Primers used in qRT-PCR validation of microarray data**

| **Index** | **Primer Name** | **Sequence 5' ---> 3'** | **Primer length** | **T_m_** | **Amplicon Length** |
| --- | --- | --- | --- | --- | --- |
| 1 | DCN + Ssc.10245.2.A1_a_at | GCATCGCTGACACCAACATT | 20 | 62.09 | 187 |
| 2 | DCN - Ssc.10245.2.A1_a_at | GTGTTGGCCAGAGAGCCATT | 20 | 62.55 | 187 |
| 3 | GRB10 + Ssc.21857.1.S1_at | TCCAAAGGCATTTGTCCTCA | 20 | 61.56 | 193 |
| 4 | GRB10 - Ssc.21857.1.S1_at | GCAGTGGTGCTTGAGTTTGC | 20 | 61.99 | 193 |
| 5 | COMMD1+ Ssc.3261.1.S1_at | CAGGCATCACAGAGGAGCTG | 20 | 62.15 | 139 |
| 6 | COMMD1- Ssc.3261.1.S1_at | GCCTCCAGCTGGTTGAAATC | 20 | 62.07 | 139 |
| 7 | DLX5 + Ssc.27249.1.S1_at | GCCAGCCCCTATCACCAGTA | 20 | 62.32 | 117 |
| 8 | DLX5 - Ssc.27249.1.S1_at | TTTCTTTGGTTTGCCGTTCA | 20 | 61.53 | 117 |
| 9 | PLAGL1 + Ssc.24770.1.S1_at | GCAAGATCTCTTCTTACGGTTTGA | 24 | 61.08 | 133 |
| 10 | PLAGL1 - Ssc.24770.1.S1_at | GCAATCTGCATCACCAGAGC | 20 | 61.94 | 133 |
| 11 | SGCE + Ssc.3772.1.A1_at | GGAAGGCGTGGAAAAGAGAA | 20 | 61.62 | 87 |
| 12 | SGCE - Ssc.3772.1.A1_at | CGGAGTTCTTTGGTAGATTTCTG | 23 | 59.31 | 87 |
| 13 | Q86TG7 + Ssc.13476.1.A1_at | CCAACATCACGTGGAGCAC | 20 | 61.19 | 93 |
| 14 | Q86TG7 - Ssc.13476.1.A1_at | GAGCGGTGGTGGTATTGGA | 20 | 61.89 | 93 |
| 15 | CD81 + Ssc.1641.1.S1_at | TTCGTCTTCTGGCTGGCTGGAG | 22 | 61.9 | 86 |
| 16 | CD81 - Ssc.1641.1.S1_at | AGGTAGAGGAGGCTGGTAGTCTGG | 24 | 62.1 | 86 |
| 17 | CDKN1C + Ssc.8871.2.A1_at | CGCACTCAGGGATTTCGG | 18 | 54.7 | 66 |
| 18 | CDKN1C – Ssc.8871.2.A1_at | CGGCGGCAGCGGTGTTGG | 18 | 63.7 | 66 |
| 19 | IGF2 + Ssc.9365.3.S1_x_at | TCGTGCTGCTATGCTGCTTACC | 22 | 60.7 | 115 |
| 20 | IGF2 - Ssc.9365.3.S1_x_at | GCCTGCCTGGAAGTCTGAAGTAG | 23 | 60.5 | 115 |
| 21 | IGF2R + Ssc.15818.1.S1_at | CGTCCGCCTGCCAGATGAAGTATG | 24 | 63.5 | 113 |
| 22 | IGF2R - Ssc.15818.1.S1_at | GCTGCCGCTGTTCTCCACCAC | 21 | 63.6 | 113 |

**Additional File 2.** These are the primer sequences used in qPCR validation of the microarray data.
